# Rosai-Dorfman disease of the oral cavity

**DOI:** 10.4322/acr.2023.463

**Published:** 2023-12-05

**Authors:** Abir Charfeddine, Mounir Omami, Marwa Garma, Ahlem Bellalah, Sameh Sioud, Jamil Selmi

**Affiliations:** 1 University Clinic of Dental Medicine, Oral Surgery Department, Oral Medicine, Monastir, Tunisia; 2 University of Monastir, Faculty of Dental Medicine, Oral Health and Oro-Facial Rehabilitation Laboratory, Monastir, Tunisia; 3 Fattouma Bourguiba University Hospital, Anatomical Pathology and Cytology Department, Monastir, Tunisia

**Keywords:** Emperipolesis, Histiocytosis, Sinus, Lymph Nodes, Maxilla

## Abstract

First described by J Rosai and R F Dorfman in 1969, Rosai-Dorfman disease (RDD) is a benign, self-limiting histiocytosis of unknown etiology. It is usually seen in the first two decades of life. The most frequent clinical presentation is painless, bilateral cervical lymphadenopathy accompanied by fever, weight loss, and an elevated ESR. However, RDD without nodal involvement is extremely rare, and the most common extranodal location is the head and neck region, mainly affecting the nasal cavity, pharynx, and paranasal sinuses. Oral location of RDD is occasional; according to our knowledge, only 17 cases of oral Rosai-Dorfman disease without lymph node involvement have been found in the literature. Because of the rarity of these isolated oral presentations, the clinical and radiological aspects need to be more studied. This article aims to present a rare case of oral Rosai-Dorfman disease without nodal involvement, detail the clinical and radiological signs, and the treatment strategy used in our patient.

## INTRODUCTION

Rosai–Dorfman disease (RDD), also known as sinus histiocytosis with massive lymphadenopathy, is a rare histiocytic disorder with unknown etiology that was first described by Destombes in 1965 and later by Juan Rosai and Ronald F. Dorfman in 1969. Previously, this entity was classified by the Working Group of the Histiocyte Society of 1987 as a non-Langerhans cell (LC) histiocytosis. RDD is part of the “R group” of histiocytoses, which includes familial RDD, sporadic RDD, and other miscellaneous non-cutaneous, non-Langerhans cell histiocytosis.^1^ The disease is rare, with an estimated prevalence of 1 in 200,000. The extranodal disease is present in 20% to 25% of cases.^2^ Although signs and symptoms vary according to the site of occurrence, the most common clinical presentation of RDD is painless, bilateral cervical lymphadenopathy accompanied by fever, elevated erythrocyte sedimentation rate, weight loss, rhinorrhea, and occasionally hepatosplenomegaly.^2^ The extranodal presentation, without nodal involvement, has been reported with a predilection for the head and neck region. The main characteristic of this disorder is the intrasinusal proliferation of benign S-100-positive histiocytes, demonstrating inflammatory cells’ emperipolesis.^2^ Various forms of treatment are suggested, ranging from local steroids to surgical excision. Long-term follow-up is advised because of the possible relapse and protracted course, associated complications such as major organ involvement, especially the kidney, which is associated with a poor prognosis or underlying immunologic abnormalities.^3-7^

We report a case of a male patient who presented Rosai–Dorfman disease in the oral cavity without any affection of lymph nodes. The clinical, histological, etiological, and treatment characteristics will be discussed in conjunction with a literature review.

## CASE REPORT

A 42-year-old male patient without medical and surgical history consulted the Department of Oral Medicine and Surgery of the dental clinic for a gingival swelling in the anterior maxilla over the last 2 months.

**Figure 1:**
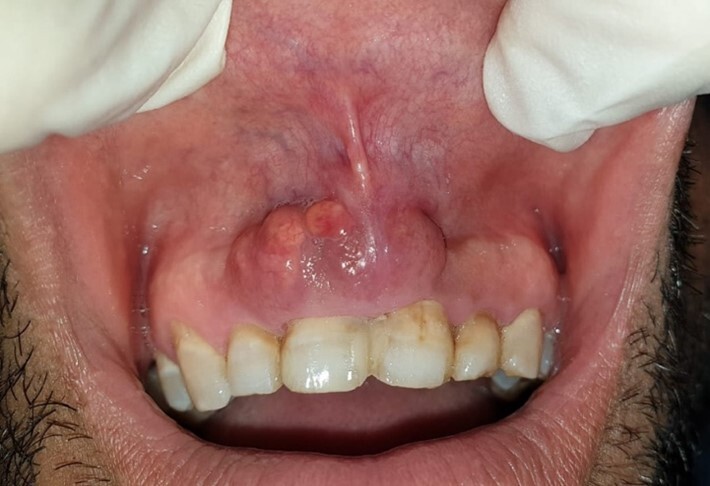
Intra oral examination. Note a polylobed, sessile nodular lesion, 3 cm long in the medio-distal direction, covered by a bluish mucosa, and purplish in places.

The patient denied using tobacco, alcohol, or medication, as well as local pain. The extra-oral examination revealed an upper lip swelling. In contrast, the intra-oral examination showed a non-tender single polylobed nodule, sessile, of 3 cm in diameter, covered with a bluish mucosa, purplish in places, firm in consistency, located in the anterior maxillary region extending to the vestibule (Figure1).

**Figure 2:**
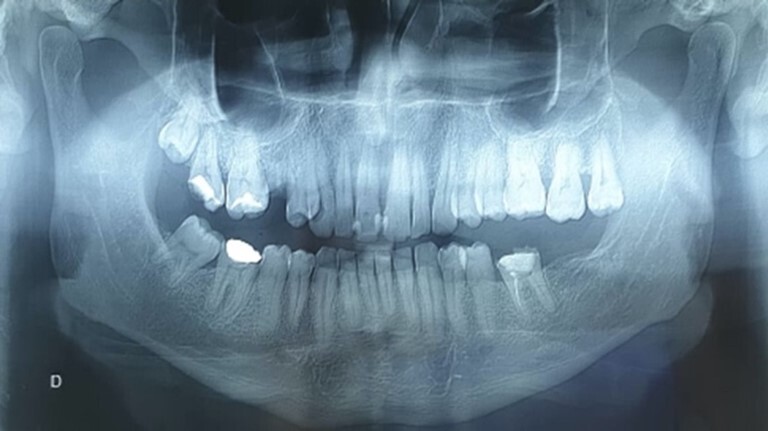
Panoramic X-ray: shows an apparent, poorly-defined anterosuperior opacity in the anterior maxilla.

The dental examination showed mobility of the upper central incisors, and the vitality test of the left upper central incisor was negative. The retro alveolar radiological examination showed a desmodontal enlargement of the upper central incisors without any other signs of bone lysis. The panoramic X-ray demonstrated no specific lesion in this area (Figure 2).

However, the computerized tomography (CT) scan showed mitted osteolysis, improperly limited, affecting the cancellous bone as well as the vestibular cortical bone in the anterosuperior region, and destruction of the floor of the nasal cavity in some sites, with the extension of the lesion towards the incisive foramen. This image extended from the mesial side of 12 to the distal side of 21, measuring 21.7 mm horizontally. The trabecular bone surrounding 11 and 21 has been destroyed, giving the appearance of floating teeth. For the soft tissues, The CT scan showed thickening of the upper lip muscles extending from the right canine to the distal side of the left central incisor.

**Figure 3:**
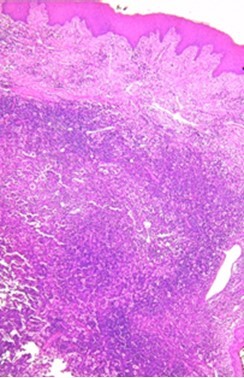
Photomicrograph of the biopsy – Gingival chorion with a dense inflammatory infiltrate rich in macrophages (H&E, 100X).

**Figure 4:**
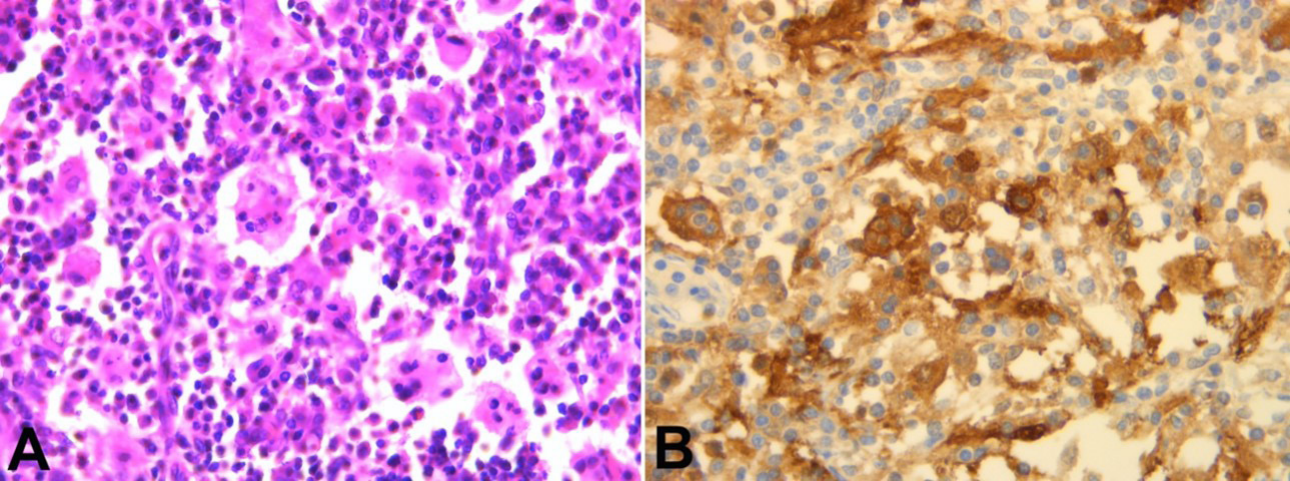
Photomicrograph of the biopsy **A** - Emperipolesis images (H&E, 400X); **B** - Intense and diffuse staining of macrophages by S100, (400X).

A gingival biopsy was performed, and the histology showed a nodular formation among the chorion with a high cell density of lymphocytes, plasma cells, eosinophilic polymorphonuclears, and especially by macrophages with abundant eosinophilic cytoplasm. These macrophages were often clustered and showed numerous images of emperipolesis (lymphocytes and some plasma cells, intact in vacuoles in the wide cytoplasm of histiocytes). The surface squamous epithelium was normal. Immunohistochemical examination shows an intense and diffuse marking of macrophages by S100. (Figures 3, 4A, and 4B).

**Figure 5:**
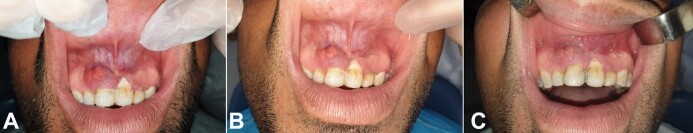
Gros view of the lesion after intralesional corticoid injection: **A** - D15, significant lesion reduction, especially at the vestibule floor; **B** - D30, decrease in gingival mass and labial swelling; **C** -D45.

Other complementary examinations, such as abdominal and chest X-ray and scintigraphy, was performed, and they did not reveal any other lesion. The complete blood count showed leukocytosis. The treatment consisted of administering an intra-lesional corticoid injection every 15 days for 2 months and a systemic corticosteroid. The clinical follow-up showed an important reduction of the lesion and lip swelling (Figures 55B, and 5C)

**Figure 6:**
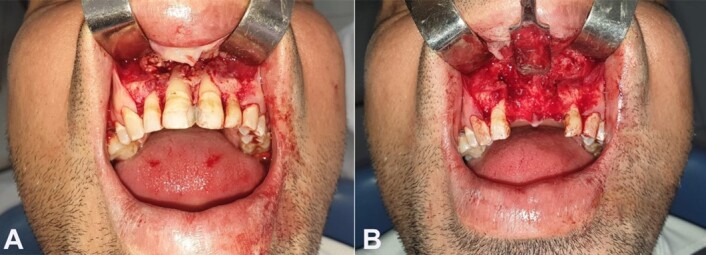
Gross view of the lesion’s surgical removal. **A** - lack of vestibular cortical bone after flap detachment; **B** - extraction of the two central incisors and total enucleation of the lesion.

Then, surgical excision was performed, with the extraction of the two maxillary central incisors, considering the mobility and the aspect of floating teeth (Figures 6A and 6B).

**Figure 7:**
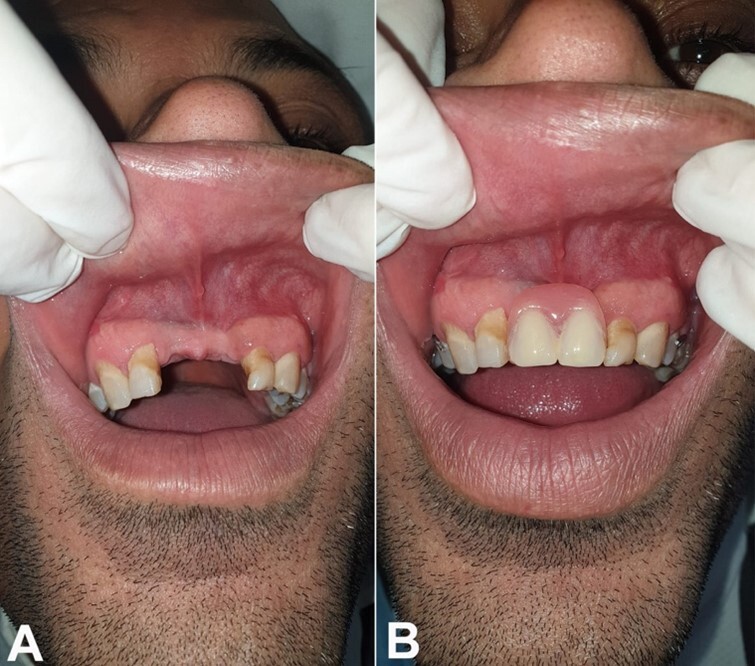
**A** - Post-surgical intraoral check-up: the disappearance of the swelling of the vestibule, with good healing of the surgical site; **B** - Placement of a removable partial denture.

A follow-up every 6 months for 2 years has been carried out until today, and the patient has no sign of recurrence. (Figures 7A, and 7B).

## DISCUSSION

As far as we know, 17 cases of oral RDD have been found in the literature without any other localization or adenopathy, and our case is the 18^th^, as shown in Table 1. Women were more affected than men. The patients' age range varied between 18 and 65 years. The maxilla was more affected than the mandible, as in our case. The most frequent patients’ complaint was pain and swelling, while others were asymptomatic, and the reason for consultation was dental mobility. Our patient’s complaint was swelling in the upper lip. Contrary to the literature, where patients presented with general symptoms, all patients with isolated oral RDD showed no fever, malaise, weight loss, or nasal obstruction. The clinical aspect of oral Rosai-Dorfman disease was not well described in the literature, with only the article by Alencar et al.,^16^ describing this lesion as a nodular, sessile, well-demarcated lesion of normal coloration and soft to palpation, associated with a reddish macule of irregular contour. This is an aspect that closely resembles the clinical aspect of our patient. Some patients had no clinical lesions, showing only tooth mobility or delayed healing of an extraction site.

**Table 1:** Characteristics of the 17 cases reported in the literature with oral RDD without nodes involvement

**Ref**	**Sex/ age (y)**	**Location**	**Clinical features**	**Radiology**	**Treatment**	**Follow-up and recurrence**
^8^	F/23	Mandible	Pain and swelling, submucosal mass	Destructive lytic and ill-defined osseous lesion	Excision	NA
^9^	F/46	Maxilla	Pain, non-healing extraction site	Expansile osteolytic process; Cortical and tuberosity resorption; involving maxillary sinus	NA	NA
^9^	F/26	Mandible	Pain, and mobility	Multiple irregular osteolytic foci; tooth extruded and its roots showed reduced bone support.		
^9^	M/26	Mandible	Nothing to report	Radiolucent lesion; cortical thinning with no significant bone expansion.		
^9^	F/38	Mandible	gingival mass	Well-demarcated expansile mass; Buccal and lingual cortical destruction	NA	NA
^10^	F/46	Maxillary ethmoid sinuses	Bilateral proptosis, sinusitis, saddle nose	NA	Excision	NA
^10^	F/47	Mandible	Swelling	NA	Excision	NA
^11^	F/44	Maxilla Nasal cavity	Pain and Swelling	NA	Excision Steroids	14 months
						No recurrence
^12^	F/29	Maxilla	Pain and swelling	NA	None	16 months
						Persistent disease
^13^	M/18	Maxilla	NA	NA	None	35 months
						Persistent disease
^14^	F/29	Maxilla	Pain and Swelling, submucosal mass	Destructive, lytic, and ill-defined osseous lesion; infiltrates in the maxillary sinus; soft tissue mass involving the maxillary sinus.	Excision	16 months No recurrence
^15^	M/12	Mandible	Pain, Swelling, vestibular edema, tenderness to percussion; rapid lesion progression	Extensive bone destruction	Partial mandibulectomy	2 years no recurrence
^16^	F/65	Maxilla	Reddish macule, telangiectasia, Nodular, sessile, lesion softened to palpation		Steroids	Monthly follow-up
^17^	F/32	Mandible	Pain and swelling, of 3 X 2 cm; and dental mobility. Dysphagia;	Well-circumscribed radiolucent lesion; mild, irregular external resorption of dental root was noted	Excision	13 months
						No recurrence
^18^	F/47	Maxilla	Dental mobility	Irregular region of bone loss with soft tissue density filling the region of the osseous defect; direct communication with the maxillary sinus.		
^19^	F/56	Maxilla	Pain and hard palate swelling involving the right genian region	NA	NA	NA
^20^	F/39	Maxilla	Pain and teeth mobility	Diffuse osteolytic image with destruction of the alveolar bone; the teeth had lost their bone support; distraction of cortical bone without the expansion of both the buccal and the palatal cortical plates; polypoid lesions and mucosal thickening of maxillary sinuses.	Steroids	5 years; Persistent disease

F: Female; M: Male; NA: Not available; y: years.

Concerning complementary examinations, all authors found no disorders in serological, hematological, and biochemical tests, unlike our patient, who presented with leukocytosis. Radiologically, all patients presented with poorly limited osteolytic lesions, destroying the cortices without expansion and extending to adjacent structures. When this lesion was related to the teeth, it resulted in a “floating tooth” appearance or caused resorption of the tooth roots.

The most commonly adopted treatment was surgical excision of the lesion, with no recurrence in all cases. The treating physicians who used only steroids noted a regression of the lesion; however, without complete remission. There is only one article,^11^ where the author prescribed steroids to the patient to reduce the size of the lesion before surgery, similar to our therapeutic strategy.

The Rosai-Dorfman disease affects all age groups, with a mean age of 20.6 years at onset.^5,7,18,21^ A literature review by Petrovic et al.,^22^ comprising 600 cases of RDD, showed that 81% were diagnosed in the first and second decades of life. This disease displays a preference for males, and most patients are of African-American descent.

About 87% of patients have cervical adenopathy, but it is known that RDD can also occur in other nodes, such as mediastinal, pulmonary hilar, retroperitoneal, axillary, and inguinal nodes.^21^

More than half of patients with lymph node involvement in RDD also demonstrate extranodal disease, with 75% of these being in the head and neck.^3,5,7,18^ The extranodal disease includes 73% in the upper respiratory tract, 50% in orbit, and 25% in salivary glands.^3,5,18^ The extranodal disease can be the initial and the only manifestation. 5 Primary involvement of the oral cavity without any other nodal/extranodal manifestations, similar to our patient, is sporadic.

The exact etiology of RDD is still unclear, but it is thought to be of genetic origin, an altered immune response, or an unidentified infectious agent, such as Herpes human virus 6, Epstein-Barr virus, parvovirus B19 (erythrovirus), zoster virus, cytomegalovirus, Brucella spp, and Klebsiella spp. In our case, no viral workup was carried out.^7,21-23^

In 85% of cases, patients with RDD are healthy, without significant symptomatology. The most common symptoms in patients with head and neck localization are fever, malaise, weight loss, nasal obstruction, rhinitis, epistaxis, anemia, leukocytosis and neutrophilia, polyclonal hypergammaglobulinemia, raised erythrocyte sedimentation rate.

Our patient presents only a leukocytosis and weight loss.^3,5,18,21^

The described clinical and radiological signs of oral involvement by RDD are nonspecific. However, some radiological signs are noted in bone involvement, as well-defined or poorly circumscribed radiolucencies, like in our case, Mixed radiolucent and radiopaque or sclerotic lesions with local destruction and extension into the adjacent soft tissues have also been described.

The histologic findings of all the cases are similar. The lesion is characterized by the proliferation of non-neoplastic histiocytes, often containing phagocytosed lymphocytes, described as lymphophagocytosis or emperipolesis, characteristic of RDD. This phenomenon is seen in both nodal and extranodal disease but is less conspicuous in extranodal lesions. At the lymph nodes, the proliferation of large histiocytes causes pronounced sinusoidal dilation and lymph node expansion. In extranodal sites, with no sinuses, the histiocytes form aggregates that resemble dilated sinuses. Other than the aforementioned cells, the presence of plasma cells and rare eosinophils are frequent. Individual cells had bland-appearing, large, round, or oval nuclei, prominent nucleoli, abundant granular, and eosinophilic staining cytoplasm, sometimes vacuolated. Varying degrees of stromal fibrosis can be observed in extranodal cases. Special stains failed to reveal any bacterial, acid-fast, or fungal organisms. By immunohistochemistry, RDD usually imparts a typical variegated dark-and-light-stained appearance. Aside from the cardinal feature of S-100 protein–positive histiocytes with emperipolesis, histiocytes are strongly positive for CD68 and negative for CD1a.^5,6,18,22,23^

The differential diagnosis of RDD is broad. It involves diseases known to have lymphadenopathies, such as tuberculosis, granulomatosis with polyangiitis, sarcoidosis, Gaucher disease, and melanoma. Identifying RDD at an extranodal site as an oral cavity without lymphadenopathy or other extranodal location leads to suspicion of different diagnoses, including Langerhans cell histiocytosis (LCH), Küttner tumor, malignant histiocytosis, Hodgkin disease, and metastatic carcinoma. The most common differential diagnosis is LCH, which shows almost the same immunohistochemical feature of S-100 protein and CD68 positivity, with the only exception of CD1a positivity of foamy histiocytes in LCH, but not in RDD, and ultrastructurally, LCH reveals characteristic rod-like Birbeck granules.^5-7,18,22^

The rarity of the disease makes the understanding of its treatment difficult. Various methods for RDD treatment are available. Treatment is not necessary in most cases. Spontaneous regression may occur, and follow-up may be adequate for asymptomatic patients who do not have involvement of vital organs. The major indication for surgery other than a biopsy is life- or function-threatening obstruction. Further options include imatinib, interferon, acitretin, thalidomide, isotretinoin, dapsone, methotrexate, rituximab, liquid nitrogen, chemotherapy, and radiotherapy. 6, 7 Reports of chemotherapy and steroids for RDD are scarce and show variable results. Nevertheless, surgery remains the most effective and definitive treatment of RDD, especially when bone involvement exists.^22^

## CONCLUSION

Although in the literature, the clinical and radiological aspect of isolated oral RDD remains poorly studied and rare, similarities with what has been published have been found. However, there is no standardized protocol for the treatment of this disease. We have adopted the combination of medical treatment to reduce the size and extent of the lesion and surgical treatment to eliminate the entire lesion.
